# Navigating Complexity: Anesthesia for Hip Fracture Surgery in an Elderly Patient With Multiple Comorbidities

**DOI:** 10.7759/cureus.85683

**Published:** 2025-06-10

**Authors:** Ahmed A Khalaf, Khaled S Abuamra, Hatem Ibrahim, Ahmad Nabil

**Affiliations:** 1 Department of Anesthesia, Dubai Hospital - Dubai Health, Dubai, ARE

**Keywords:** chronic kidney disease, elderly patients, interdisciplinary collaboration, intrathecal clonidine, perioperative care, spinal anesthesia

## Abstract

This case report delineates the anesthetic management of an 80-year-old male undergoing surgical fixation of a left femoral neck fracture, complicated by coronary artery disease, stage 5 chronic kidney disease, and type 2 diabetes mellitus. Utilizing spinal anesthesia, the anesthetic team achieved a successful perioperative outcome through rigorous preoperative optimization, precise intraoperative management, and comprehensive postoperative care. The absence of intraoperative complications highlights the efficacy of regional anesthesia in mitigating risks in high-risk elderly patients. This report underscores the pivotal role of tailored anesthetic strategies and interdisciplinary collaboration in optimizing outcomes for complex hip fracture cases, contributing to the evidence supporting regional anesthesia.

## Introduction

Hip fractures in elderly patients pose significant perioperative challenges due to age-related physiological decline and the burden of comorbidities, which increase the risk of adverse outcomes [1]. Anesthetic management requires careful consideration of cardiovascular, renal, and metabolic factors while minimizing complications such as postoperative delirium and cardiopulmonary events [2]. Spinal anesthesia has emerged as a preferred approach, offering reduced risks compared to general anesthesia [3]. This case report describes the anesthetic management of an elderly patient with multiple comorbidities undergoing hip fracture repair, illustrating the strategic application of spinal anesthesia and multidisciplinary care to achieve a favorable outcome.

## Case presentation

**Figure 1:**
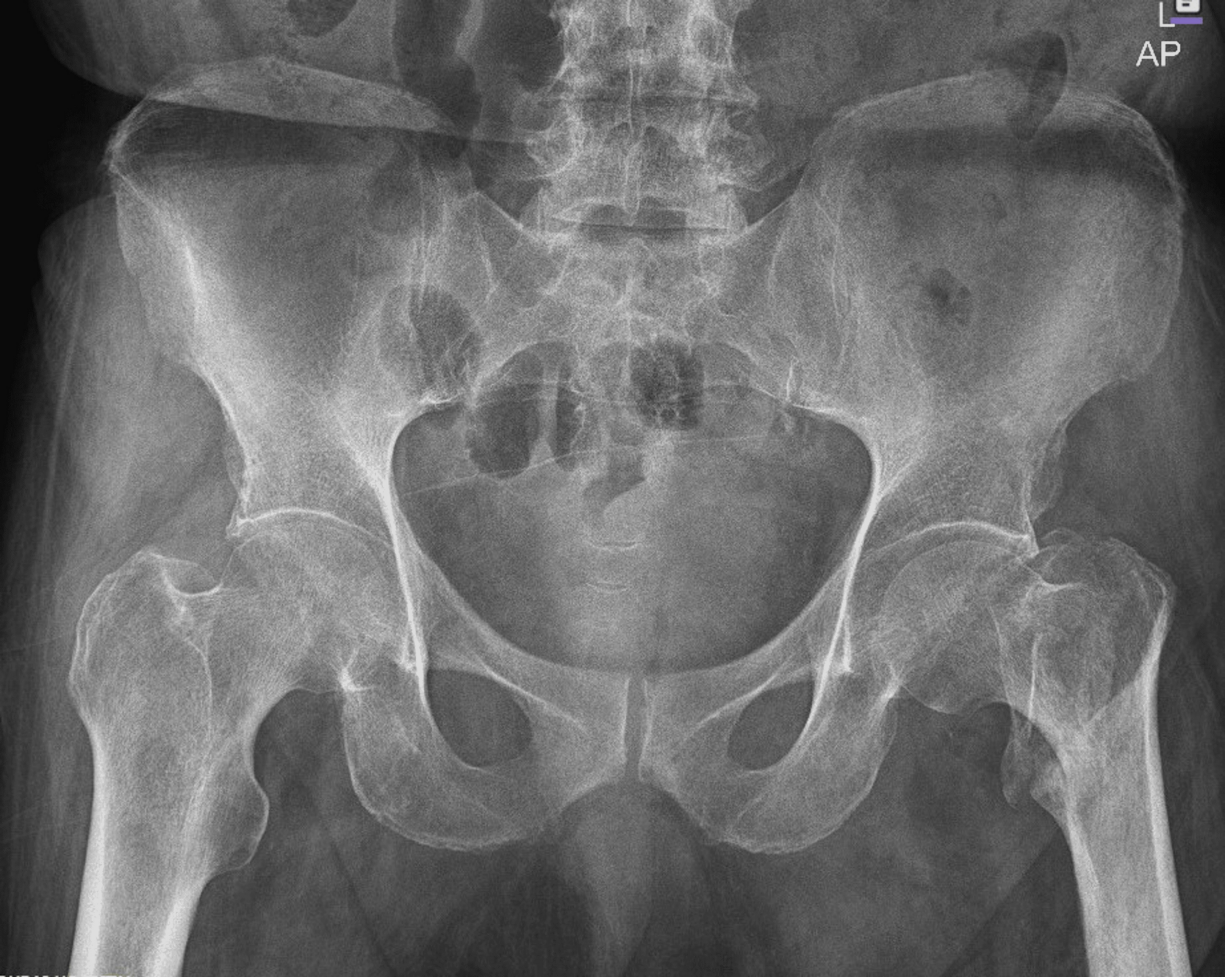
X-ray pelvis showing the mildly displaced intertrochanteric fracture of the left femur.

An 80-year-old male presented to the Emergency Department following a fall resulting in a closed left femoral neck fracture. He was scheduled for closed reduction and internal fixation with a trochanteric femoral nail advanced (TFNA) as confirmed by X-ray pelvis showing a "mildly displaced intertrochanteric fracture of left femur" (Figure 1). His medical history included coronary artery disease with prior coronary stenting (echocardiography revealed preserved systolic function (ejection fraction 55-60%), Grade I diastolic dysfunction, and no valvular lesions, regional wall motion abnormalities, or pulmonary hypertension, indicating low perioperative risk for anesthesia), poorly controlled hypertension, end-stage renal disease requiring twice-weekly hemodialysis, and well-controlled type 2 diabetes mellitus. Additional conditions included benign prostatic hyperplasia, bilateral renal cortical cysts, and cystitis. His medications comprised bisoprolol, aspirin, enoxaparin (substituted for clopidogrel preoperatively), insulin, ezetimibe, and topical beta-sitosterol, with no reported drug allergies.

Preoperative Assessment

**Figure 2:**
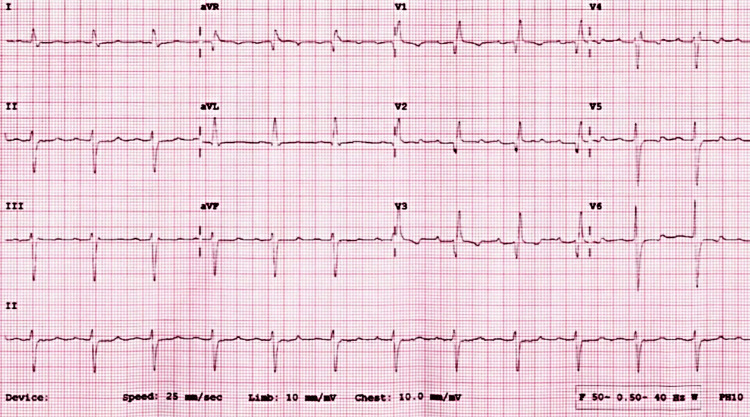
ECG showing the sinus rhythm with the right bundle branch block.

**Figure 3:**
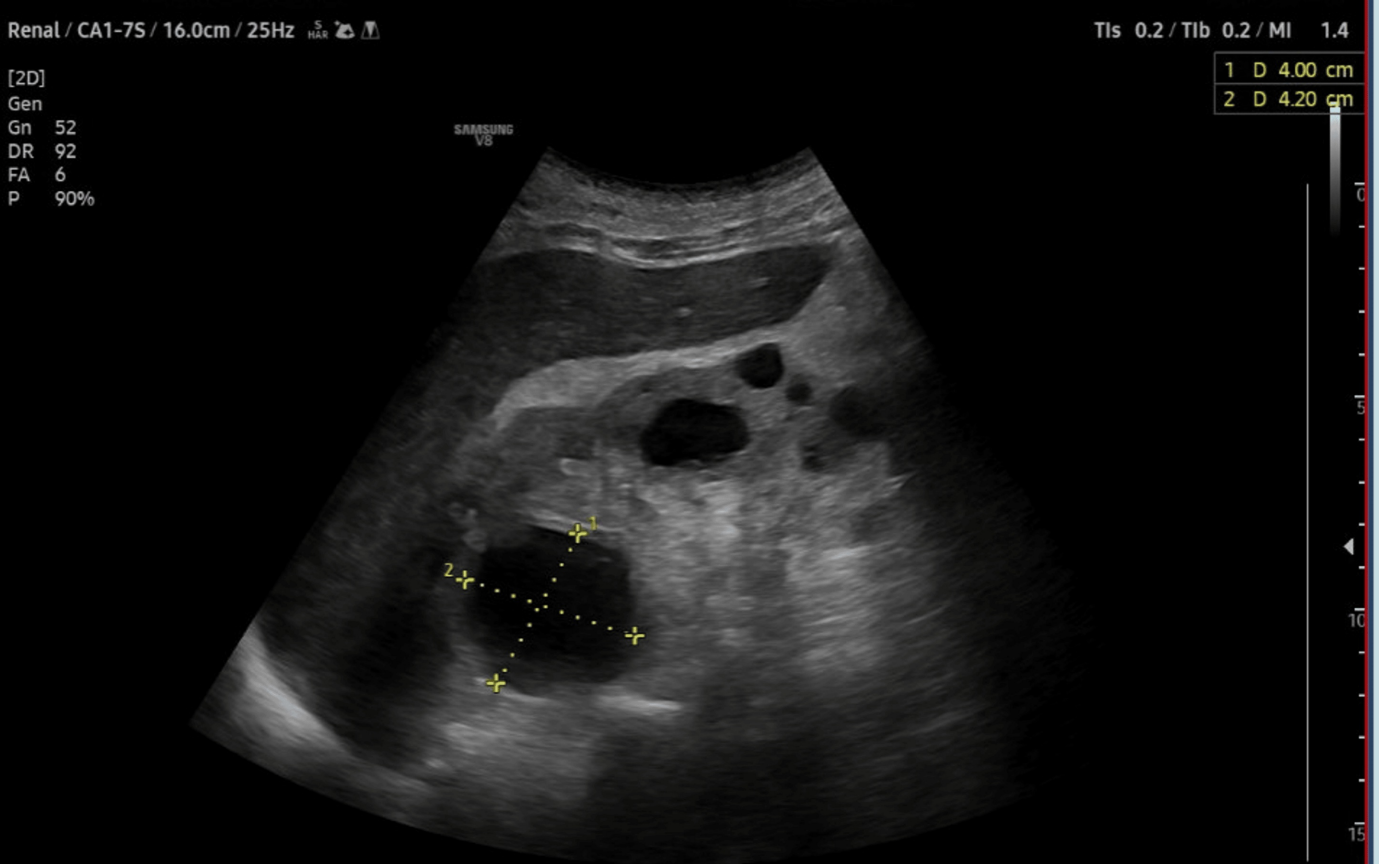
Renal ultrasound images showing renal cortical cysts.

**Figure 4:**
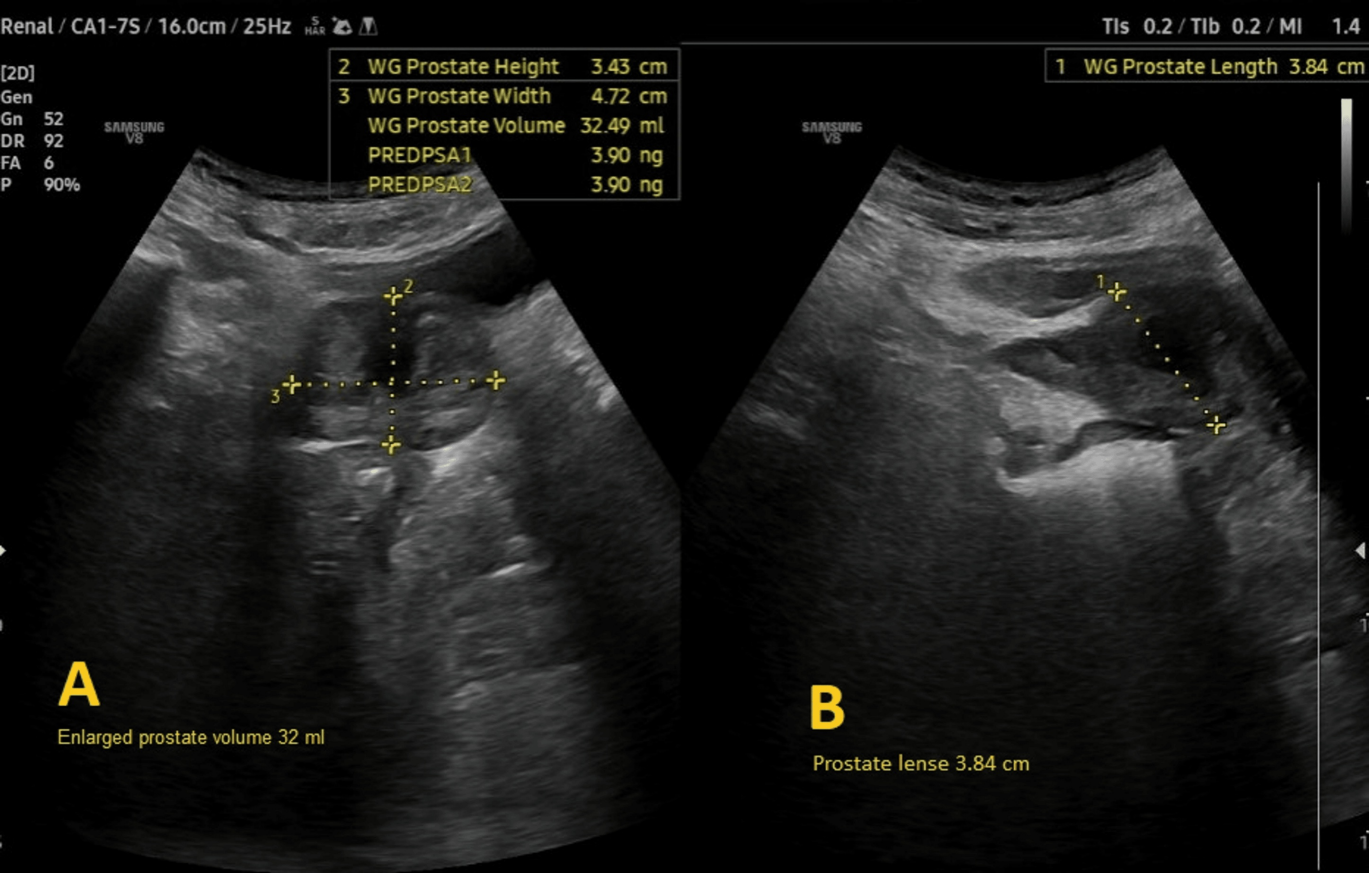
Pelvis ultrasound images showing an enlarged prostate.

**Figure 5:**
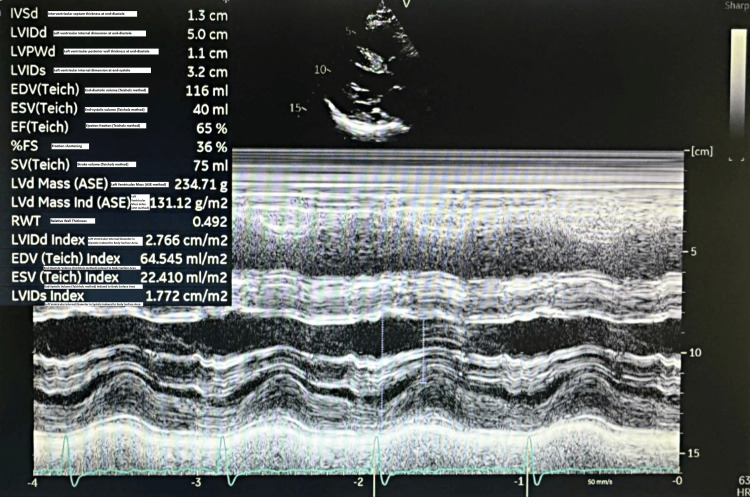
Transthoracic echocardiogram visualizing the normal left ventricular function and structure.

Preoperative evaluation classified the patient as American Society of Anesthesiologists (ASA) physical status 3, reflecting severe systemic disease. The Revised Cardiac Risk Index (RCRI) score indicated Class III, with a major adverse cardiac event risk exceeding 10.1% [4]. Electrocardiography showed sinus rhythm with right bundle branch block (Figure 2), while chest radiography was unremarkable. Airway assessment revealed a Mallampati Class II score, full cervical mobility, and removable partial dentures. Laboratory findings (Table 1) included elevated serum creatinine (4.98 mg/dL), mild hyperkalemia (5.7 mmol/L), mild anemia (hemoglobin of 11.6 g/dL), and a normal international normalized ratio (INR: 1.00). Imaging confirmed the femoral neck fracture, with ultrasonography noting renal cysts (Figure 3) and prostatic enlargement (Figure 4). An echocardiogram was normal (Figure 5). Cardiology consultation advised continuing bisoprolol, resuming aspirin postoperatively, and bridging anticoagulation with enoxaparin, discontinued 24 hours preoperatively. Recent hemodialysis stabilized electrolytes, optimizing the patient for surgery.

**Table 1:** Preoperative laboratory investigations. CKD-EPI: chronic kidney disease epidemiology collaboration; eGFR: estimation of glomerular filtration rate; INR: international normalized ratio

Component	Result	Ref Range & Units
Creatinine	4.98 (High Panic)	0.70-1.20 mg/dL
eGFR (CKD-EPI)	11.1 (Low)	>60 mL/min/1.73 m²
Potassium	5.7 (High)	3.3-4.8 mmol/L
Hemoglobin, Blood	11.9 (Low)	13.0-17.0 g/dL
INR	1.00	0.8-1.1

Anesthetic Management and Intraoperative Course

**Figure 6:**
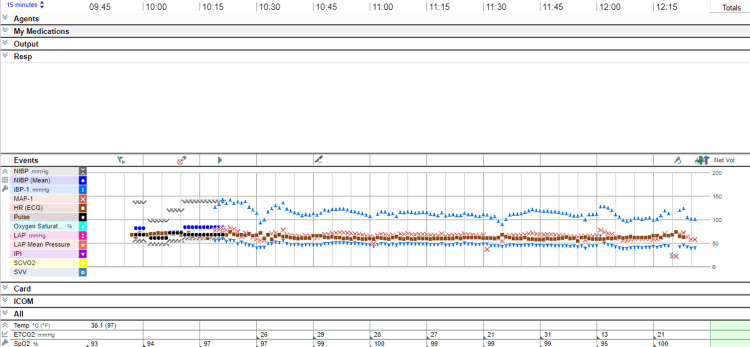
Intraoperative vitals. A screenshot of an Epic system that uses the tab-separated value (TSV) format from the patient's real record.

Given the patient’s cardiovascular and renal comorbidities, spinal anesthesia was selected to minimize risks associated with general anesthesia, including postoperative cognitive dysfunction, hemodynamic instability, nausea and vomiting, and opioid need postoperatively [5]. Contingency plans included peripheral nerve blocks, epidural anesthesia, or general anesthesia. Sedation using midazolam 1 mg and dexmedetomidine 6 mcg intravenously was given before positioning for spinal anesthesia. Spinal anesthesia was performed at the L3-L4 interspace in the right lateral decubitus position using a 22-gauge needle after two attempts. Spinal anesthesia was tolerated well by the patient. The patient was kept in lateral position for 10 minutes after injection of spinal medicines to improve block selectivity, reduce block extension, and improve the patient's hemodynamics. A combination of 2 mL hyperbaric bupivacaine 0.5% (10 mg) and 30 mcg clonidine achieved a T8 sensory level and complete bilateral motor block (Bromage score 3) [6]. The patient was positioned supine on a fracture table with padded pressure points and monitored with continuous electrocardiography, pulse oximetry, invasive arterial blood pressure, and intermittent tympanic temperature (Figure 6).

**Figure 7:**
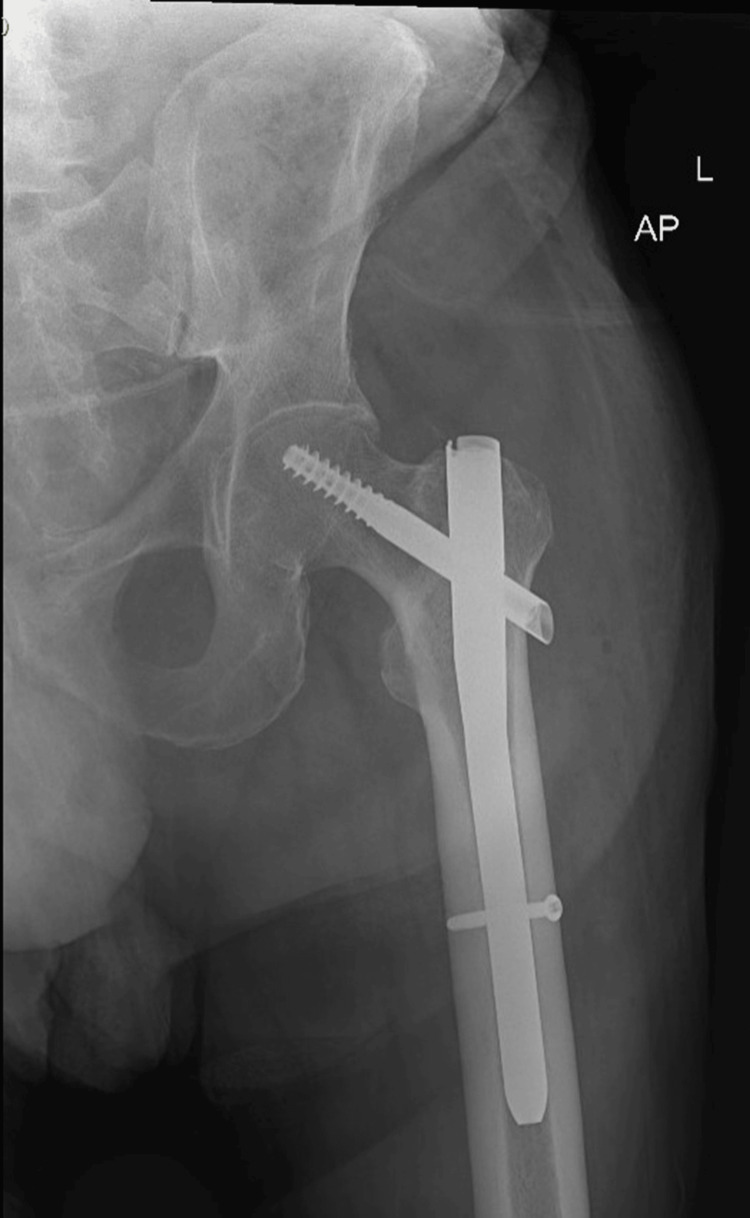
Left hip AP view showing internal fixation with good alignment.

The surgical procedure, involving closed reduction under fluoroscopy and TFNA fixation, lasted 95 minutes (Figure 7). Sedation was maintained through the procedure with a second dose of 6 mcg of dexmedetomidine. Hemodynamic stability was maintained with minimal intravenous phenylephrine (5-20 mL/hour at 100 mcg/mL). Intraoperative fluids comprised 500 mL sodium chloride and 200 mL 5% albumin in normal saline, with 1.5 g cefuroxime administered for prophylaxis. Vascular access was secured via a 20-G peripheral intravenous line (left basilic vein) and a right radial arterial line, removed post-procedure. Estimated blood loss was minimal (100 mL), and no blood transfusions were required. The procedure concluded without intraoperative complications.

Postoperative Course

The patient was monitored in the post-anesthesia care unit (PACU) for one hour. Vital signs remained stable: temperature of 36.4°C, pulse of 71-73 beats per minute, blood pressure of 95/46-108/50 mmHg with infusion of 250 mL of normal saline during PACU stay, and oxygen saturation of 96-97% on room air. Pain scores (visual analogue score) were 0, indicating effective analgesia, and a Modified Aldrete score of 9 confirmed readiness for discharge. Residual motor block (Bromage score 3) and reduced sensation below the umbilicus persisted, consistent with the spinal block. The bed head was elevated to 30 degrees for comfort. The patient restored full motor recovery after 45 minutes from discharge from the PACU; meanwhile, pain was fully controlled for eight hours following discharge from the PACU. Given the patient's co-morbidities, the patient was admitted to a high dependency unit (HDU) before the procedure. The patient was discharged from the PACU to the HDU post procedure. The patient had an uneventful post-procedure course and discharged home after 72 hours.

## Discussion

This case highlights the efficacy of spinal anesthesia in managing elderly patients with significant comorbidities undergoing hip fracture surgery. Regional anesthesia aligns with evidence demonstrating reduced postoperative delirium, respiratory complications, and hemodynamic instability compared to general anesthesia. The use of clonidine as an adjuvant extended the duration of the spinal block, minimizing intraoperative analgesic requirements [7]. Spinal anesthesia also reduces the risk of postoperative complications, such as deep vein thrombosis and pulmonary embolism, which is particularly critical in elderly patients with cardiovascular comorbidities, as seen in this case. Additionally, regional anesthesia has been associated with shorter hospital stays and lower postoperative opioid requirements, which was advantageous for this patient with chronic kidney disease, where opioid sparing helped prevent exacerbation of renal dysfunction.

Preoperative optimization, including hemodialysis and anticoagulation management, effectively mitigated renal and cardiovascular risks [8]. Hemodialysis stabilized electrolytes, addressing the patient’s hyperkalemia and reducing the risk of fluid overload, which is critical in end-stage renal disease. The anticoagulation strategy, involving enoxaparin bridging discontinued 24 hours preoperatively, balanced bleeding and thrombotic risks in the context of coronary artery disease. This meticulous preoperative planning was essential given the patient’s high cardiac risk, as indicated by the RCRI Class III status, which is associated with increased perioperative morbidity in hip fracture surgery.

Fluid administration was carefully titrated to avoid overload in the context of chronic kidney disease, and minimal blood loss reflected precise surgical and anesthetic techniques [9]. The use of 5% albumin and sodium chloride, guided by invasive arterial monitoring, maintained hemodynamic stability without compromising renal function. Goal-directed fluid therapy, as applied here, has been shown to reduce postoperative acute kidney injury in elderly patients with comorbidities. The minimal blood loss (100 mL) facilitated by spinal anesthesia’s hemodynamic stability further reduced the need for transfusions, aligning with evidence supporting regional anesthesia for precise surgical interventions [3].

Postoperative HDU monitoring was essential given the patient’s RCRI Class III status, with the Modified Aldrete score guiding safe PACU discharge. The absence of postoperative delirium or cardiopulmonary complications reinforces the benefits of spinal anesthesia in reducing postoperative cognitive dysfunction in elderly patients. The interdisciplinary collaboration between anesthetists, orthopedic surgeons, cardiologists, and nephrologists was instrumental in achieving a successful outcome, reinforcing the value of coordinated care in complex cases. Multidisciplinary care pathways have been shown to improve 30-day mortality rates in hip fracture patients with multiple comorbidities by ensuring seamless communication and tailored interventions.

In summary, this case underscores the advantages of spinal anesthesia, supported by robust preoperative optimization and interdisciplinary collaboration, in achieving optimal outcomes for high-risk elderly patients. The integration of clonidine, precise fluid management, and coordinated care aligns with current evidence and highlights the importance of tailored anesthetic strategies in mitigating perioperative risks.

## Conclusions

Spinal anesthesia, supported by thorough preoperative optimization and vigilant postoperative monitoring in a high-dependency setting, proved highly effective for hip fracture fixation in this elderly patient with multiple comorbidities. This case is considered to be a preliminary observation that highlights an area for further investigation, which reinforces the advantages of regional anesthesia and multidisciplinary collaboration in optimizing perioperative outcomes in high-risk populations coming for hip surgery. The use of opioid free anesthesia represented by intrathecal clonidine needs further studies in order to demonstrate the ideal dosage and the exact extension of block and analgesic duration related to its use.
